# Icaritin suppresses multiple myeloma, by inhibiting IL-6/JAK2/STAT3

**DOI:** 10.18632/oncotarget.3399

**Published:** 2015-03-16

**Authors:** Shicong Zhu, Zhihua Wang, Zijian Li, Hongling Peng, Yunya Luo, Mingyang Deng, Ruijuan Li, Chongwen Dai, Yunxiao Xu, Sufang Liu, Guangsen Zhang

**Affiliations:** ^1^ Division of Hematology, Institute of Molecular Hematology, The Second Xiang-ya Hospital, Central South University, Changsha, Hunan, China; ^2^ Division of Hematology, The First Affiliated Hospital, Lanzhou University, Lanzhou, Gansu, China

**Keywords:** icaritin, multiple myeloma, antitumor activities, IL6/JAK2/STAT3 signaling

## Abstract

Icaritin is an active prenylflavonoid derived from Epimedium genus, a traditional Chinese medicine. Icaritin has a wide range of pharmacological and biological activities, including cardiovascular function improvement, hormone regulation and antitumor activity. Here, we investigated the effect of icaritin on multiple myeloma (MM) *in vitro* and *in vivo*. Icaritin inhibited cell growth of MM cell line and primary MM cells. In contrast, icaritin had low or no cytotoxic effect on normal hematopoiesis. We also demonstrated that in MM xenograft mouse models, icaritin suppressed tumor growth and decreased serum IL-6 and IgE levels, but did not show adverse reactions such as body weight loss. The anti-MM activity of icaritin was mainly mediated by inhibiting IL-6/JAK2/STAT3 signaling. We suggest that icaritin can be further tested in clinical trials in MM.

## INTRODUCTION

Multiple myeloma (MM) is characterized as malignant plasma cell proliferation, and is the second most frequent hematological malignancy that accounts for 10% of hematological tumors [1]. MM primarily affects older individuals (the median age >65 years). In the last decades because myeloma treatment is developing rapidly, including thalidomide, bortezomib, lenalidomide, and autologous stem cell transplantation, the outcome of MM has significantly improved. However, MM remains incurable disorder due to chemotherapy resistance or disease-refractory [2]. Therefore, the search for new and effective therapeutic agents for MM treatment is urgently required.

A new approach in treating cancer is the awareness of the important pharmacological action of many natural products such as lycopene, curcumin, EGCG, resveratrol etc [3, 4]. Current research has established significant roles for natural products in receptor binding, proliferation signaling modulation, pro-apoptosis pathway and targeting various oncoproteins without affecting normal cells [5].

Icaritin, a hydrolytic product of icariin, is a component of the traditional Chinese herbal medicine Epimedium Genus. It also is one of the principle active prenylflavonoids. Previous studies showed that icaritin had a wide range of pharmacological and biological activities, including stimulation of neuronal and cardiac differentiation [6, 7], enhancement of osteoblastic and suppression of osteoclastic differentiation and activity [8] and inhibited the growth of hematological malignancies [9, 10]. As natural flavonoids, icaritin possesses estrogen-like activity. It had been documented that icaritin exhibited estrogen-like activity in estrogen receptor-positive breast cancer MCF-7 cells at sub-micromolar concentration [11]; while at micromolar levels, icaritin might inhibit the growth of prostate cancer PC-3 cells [12], and suggesting icaritin may function as an estrogen receptor modulator in regulating cell growth. Some data indicated that both estrogen α receptor and estrogen β receptor could be detected in multiple myeloma cell lines, such as KMM-1, KMS-11, KMS-18, KMS-20 and U266 [13], suggesting the dysregulation of interaction between estrogen and estrogen receptor may be involved in the pathogenesis of MM. As stated above, the median age of patients with MM is 65 years and approximately 80% develop substantial skeletal dysfunction including diffuse osteopenia, focal lytic bone lesions, pathologic fractures and so on [14]. Numerous studies of bone histology have shown that MM is characterized by excessive resorption of bone and inhibition of bone formation [15]. These changes, to a certain extent, are mimic the changes of the aged, especially in menopausal women, suggesting an imbalance of estrogen levels may contribute to the pathogenesis of MM. Thus whether icaritin, as a plant estrogen, may target the estrogen receptor of MM cells, and play anti-MM activities, it constitutes our study imagine.

We have previously shown that icaritin can effectively inhibit chronic myeloid leukemia (CML) cells growth and induce CML cells apoptosis via the mechanisms involved in MAPK/ERK/JNK and JAK2/STAT3/AKT signaling [10]. The STATs are reported as latent cytoplasmic transcript factor in response to all cytokine driven signaling [16]. STAT3 is often constitutively activated in many human cancer cells such as multiple myeloma, leukemia, lymphoma, and solid tumors [16, 17], which confers myeloma cells resistance to apoptosis through regulation of the anti-apoptotic protein Bcl-xL [18]. It has been established that interleukin-6 (IL-6) is the most important survival and growth factor in myeloma cells [19] and regulated at least 3 pathways, namely JAK2/STAT3, MAPK/ERK, and PI3K/AKT [20], thus targeting IL-6-mediated signaling pathways is a promosing therapeutic strategy in MM [21–23]. On account of the effects of icaritin in disturbing the signaling of JAK2/STAT3/AKT on CML cells, we try to determine whether icaritin is able to target the IL-6/JAK2/STAT3 driven-signalings on MM cells. In present study, we investigated the effects of icaritin against MM activities on myeloma cell line-U266, primary bone marrow mononuclear cells (BMMCs) and CD138^+^ cells from MM patients *in vitro* and mouse xenograft model *in vivo* to firmly determine the role of icaritin in anti-myeloma activity, and elucidate partly the mechanism of icaritin in anti-MM effects. Our results suggest that icaritin is a specific inhibitor of IL-6/JAK2/STAT3 signaling and may represent an alternative therapeutic strategy for the treatment of refractory MM.

## RESULTS

### Icaritin suppresses proliferation of both U266 cells and primary MM cells

**Figure 1:**
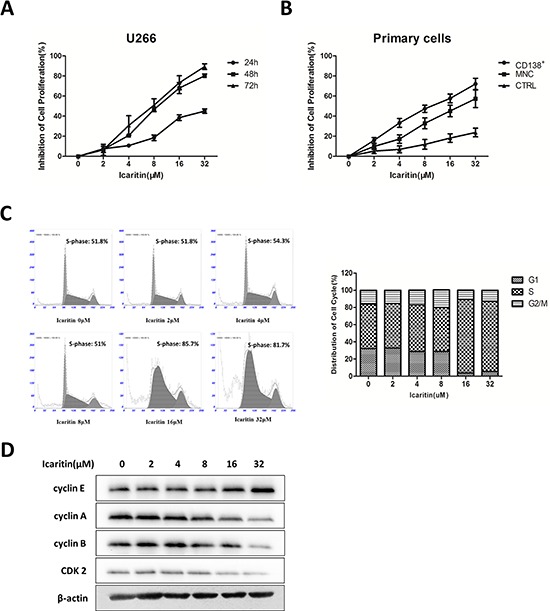
Icaritin (ICT) inhibits U266 cells proliferation and arrests cells cycle progress by downregulate cyclin-related proteins **A.** Dose-, or time-response curves for the effects of icaritin in proliferation inhibition on human MM U266 cell line. The values represent mean ± SD of triplicate cultures. **B.** Effect of icaritin on the growth inhibition on fresh CD138^+^ MM cells from MM patients (*n* = 14) or fresh primary cells (BMMCs) from MM patients (*n* = 28), and BMMCs from normal controls (*n* = 11). Mean ± SD. **C.** Icaritin-induced S-phase arrest on U266 cells after exposed to Icaritin (0–32 μM) for 48 h. Mean ± SD (*n* = 3). **D.** Icaritin down-regulates cyclin A, cyclin B and CDK2, and up-regulates cyclin E proteins on U266 cells (Western blot result).

To explore the effects of icaritin on growth of MM cells, we treated cells with icaritin at different concentrations for 24, 48 and 72 hours and assessed the cell viability by MTT assay. Our results showed that icaritin effectively inhibited U266 cells proliferation with dose- or time-dependent manner. The IC_50_ value of icaritin were 36.63 μM (24 h), 10.05 μM (48 h) and 8.60 μM (72 h) (Figure 1A). We also found that icaritin exhibited significantly growth-inhibiting effect on CD138^+^ MM cells (*n* = 14, IC_50_ = 10.31 μM, 48 h), corresponding to primary MM cells from BMMCs (*n* = 28, IC_50_ = 20.91 μM, 48 h) and BMMCs of normal controls (*n* = 11, IC_50_ = 240.5 μM, 48 h) (Figure 1B).

### Icaritin results in S phase arrest by targeting cyclin-related proteins and CDK2 on U266 cells

To further determine the proliferation-inhibiting effect of icaritin on U266 cells and explore involved signaling pathway, we measured cell cycle distribution of U266 cells and the changes of cell cycle-regulating proteins under icaritin treatment. The results showed icaritin lead to significantly S phase arrest in a dose-dependent manner (Figure 1C). To investigate the molecules affected by icaritin, we examined the expression levels of several S phase-related proteins. Cdk2-cyclin E control G1 entry into S phase [24, 25]. Upon entry into S phase, cyclin E is rapidly degraded by the ubiquitin-proteosome system. Cdk2-cyclin A regulates S phase progression and the accumulation and activation of Cdc2-cyclin B at the G2/M transition [26]. Icaritin evidently reduced cyclin A, cyclin B and CDK2 expression, and upregulated the expression of cyclin E (Figure 1D). These results suggest that icaritin could induce S phase arrest in U266 cells.

### Icaritin induces U266 cells and primary MM cells apoptosis by caspases activation and Bcl-xL signaling interference

**Figure 2:**
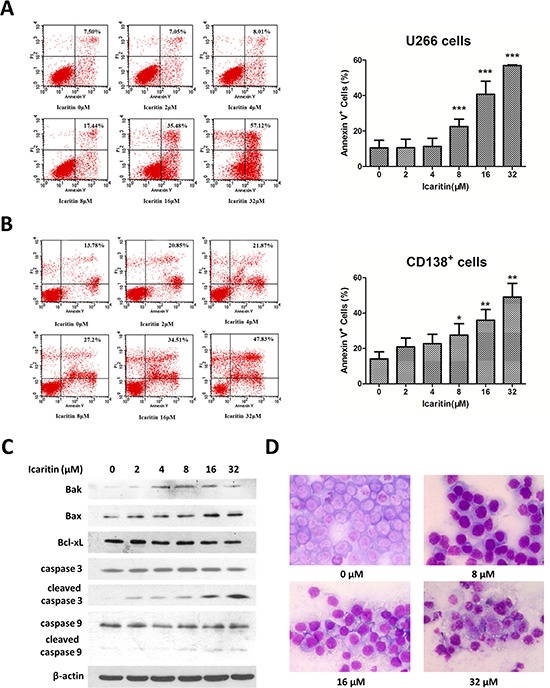
Icaritin induces U266 cells or CD138+ primary MM cells apoptosis **A and B.** U266 cells and CD138^+^ primary MM cells were treated with increasing concentrations of icaritin for 48 h, which was followed by analysis of apoptosis by staining with PI and Annexin-V FITC. Annexin-V positive cells were measured by flow cytometry. Columns represent the average percent of Annexin V positive cells from more than 3 independent experiments, which are shown as the mean ± SD. Asterisks (***) indicates statistically significant (*p* < 0.001) differences. Asterisks (**) or (*) represents statistically significant differences (*p* < 0.01) or *p* < 0.05, respectively. Representative images are shown in the left panel. **C.** Effects of icaritin on casepase 3, caspase 9, bak, bax, bcl-xl expression (western blot results). β-actin is used as loading control. **D.** Morphological features for apoptosis in untreated and icaritin-treated U266 were revealed by Wright-Giemsa staining under light microscope (Carl Zeiss Axio Scope A1) at 400× magnification.

To confirm whether the anti-tumor activity of icaritin is associated with apoptosis, we assessed morphologic changes in icaritin-treated cells. U266 exposed to different concentrations of icaritin for 48 h displayed morphologic characteristics of apoptosis such as condensation of nuclear, membrane blebbing, as revealed by light microscope with Wright-Giemsa staining (Figure 2D). Externalized phosphatidylserine (PS), an indicator of early apoptosis, as revealed with the annexin V-FITC staining, was remarkably increased both in icaritin-treated U266 cells and CD138^+^ MM cells (Figure 2A, 2B). To evaluate the molecular events of apoptosis arising from icaritin treatment, western blot was performed for detecting the expression of caspase 3, caspase 9, Bax, Bak and Bcl-xL proteins. As shown in Figure 2C, icaritin significantly upregulated the expression of Bak and Bax and inhibited Bcl-xL expression with dose-dependent manner. Following increased icaritin concentration, caspase 3 and caspase 9 were cleaved and activated. These results suggest that icarritin induced MM cells apoptosis is involved in caspases pathway.

### Icaritin inhibits IL-6/JAK2/STAT3 signaling in U266 cells

**Figure 3:**
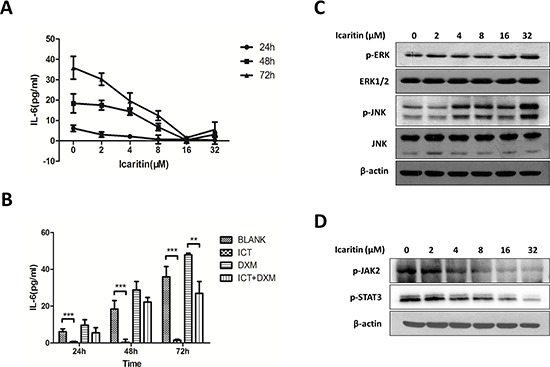
Anti-MM effect of icaritin was associated with decreasing IL-6 level and inhibiting the downstream signalings **A.** Icaritin decreased significantly the autocrine IL-6 levels at time-dependent (24 h, 48 h, 72 h) or dose-dependent (2–32 μM) manner in U266 cells. Results represent the mean of three independent experiments. **B.** Effect of icaritin (16 μM), dexamethasone (10 μM) on autocrine IL-6 in U266 cells. Cells were treated with the different agents for 24, 48 or 72 hours. Data portrayed are the mean of separate experiments with the SD showed. Statistical significance was determined by 2-tail Student's *t*-test. **p < 0.01; ***p < 0.001. **C.** Icaritin (2–32 μM) up-regulated the expression of p-JNK and p-ERK in U266 cells (48 h) (western blot results). **D.** U266 cells were treated with icaritin at indicated doses for 24 h, p-JAK2 and p-STAT3 were detected by Western blot.the results showed Icaritin inhibited the expression of p-JAK2 and p-STAT3.

It has been shown that IL-6-mediated autocrine loop in U266 cells was involved in the resistance to dexamethasone (DXM)-induced apoptosis [27]. Baicalein, a major flavonoid derived from *Scutellaria radix*, was able to inhibit IL-6 expression in U266 cells [20]. To explore the potential mechanisms regulating the effects of icaritin on U266 cells, and determine if anti-MM activity of icaritin is related to the inhibition of IL-6 mediated autocrine loop, we examined several major oncogenic signaling pathways, including IL-6, JAK2, STAT3, and two members of mitogen-activated protein kinase (MAPK) family: JNK and ERK. Our results demonstrated that icaritin was able to reduce significantly the levels of IL-6 in cultured U266 cells supernatant with dose- or time-dependent manner (Figure 3A). Of note, compared with DXM-treatment, icaritin exhibited a persistent inhibition on IL-6 levels and reversed the DXM-resistance of U266 cells to a certain extent (Figure 3B). Although icaritin had no effect on total JNK and ERK proteins, evidently it up-regulated expression of phospho-JNK (p-JNK) and phospho-ERK (p-ERK) (Figure 3C).

We further evaluated the potential effects of icaritin on the status of JAK2 and STAT3, which are frequently activated in myeloma cells. Our initial results showed that icaritin treatment inhibited the expression of phospho-STAT3 (p-STAT3) and phospho-JAK2 (p-JAK2) proteins in U266 cells in dose-dependent way (Figure 3D). Collectively, these data suggest that anti-tumor activity and pro-apoptotic effect of icaritin on human U266 cells is associated with the mechanism involved in targeting IL-6/JAK2/STAT3 signaling pathway.

### Icaritin-induced apoptosis is regulated by JAK2/STAT3 signaling in U266 cells

**Figure 4:**
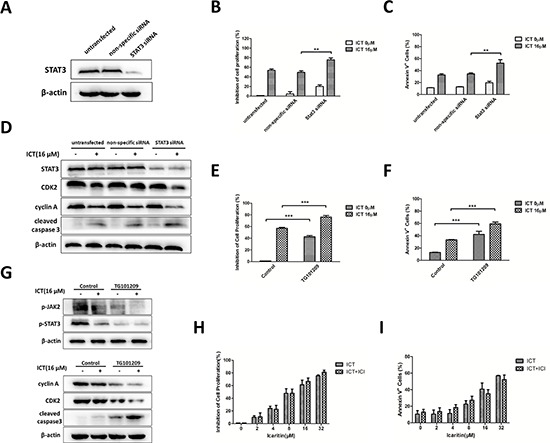
Icaritin exerts anti-MM activity via suppressing JAK2/STAT3 signaling pathway, not associated with estrogen receptors **A.** U266 cells were transfected with STAT3-directed siRNA and non-specific siRNA (control), the success of STAT3 silencing was confirmed by western blotting. **B and C.** U266 cells were transfected with STAT3 siRNA or non-specific siRNA, and then treated with or without 16 μM icaritin for 48 h. Cell proliferation was analyzed by MTT and cell apoptosis was assessed by Annexin V-FITC/PI staining with flow cytometry. The data represent the mean ± SD (*n* = 3), **p < 0.01. **D.** Effect of silencing STAT3 on the expression of cell cycle and apoptosis-related proteins. These figures are from three separate experiments. **E and F.** U266 cells were treated with icaritin (16 μM) or co-treated with icaritin (16 μM) and TG101209 (2 μM) for 48 h. The cell proliferation and apoptosis were detected as mentioned-above methods. The data represent the mean ± SD of three independent experiments. ***p < 0.001. **G.** Icaritin alone or combination with TG101209 treated U266 cells for 24 h for evaluating the expression of p-JAK2, p-STAT3 and STAT3 or for 48 h for assessing the expression of cell cycle- and apoptosis-related proteins by western blotting. Representative images were shown. **H and I.** U266 cells were pretreated with ICI182, 780 (1 μ) for 4 h, and exposed to various dose of icaritin (0–32 μ) for additional 48 h. Inhibition of cell proliferation was evaluated by MTT, and cells apoptosis was detected by Annexin V-FITC/PI staining combined flow cytometry. The data represent the mean ± SD (*n* = 3). There are no statistically significant differences between icaritin and icaritin+ICI182, 780 groups.

To further investigate whether JAK2 or STAT3 signaling activity directly affects the biological effects of icaritin in MM cells, U266 cells were transfected with siRNA against STAT3 or a control vector (non-specific siRNA). Transfected cells were confirmed by western blot (Figure 4A). After transfection, the effect of icaritin for proliferation-inhibiting in U266 cells was enhanced (Figure 4B), and icaritin-induced apoptosis was significantly increased compared with non-specific siRNA group (Figure 4C). More interestingly, several STAT3-regulated proteins important for tumor cell growth and apoptosis, such as CDK2, cyclin A or caspase 3, exhibited a down-regulated expression and cleaved activation respectively (Figure 4D) in STAT3-knockdown U266 cells.

STAT3 is activated by soluble tyrosine kinases JAKs. To explore whether JAK2 play a key role in the anti-tumor effects of icaritin on U266 cells, we treated U266 cells with icartin in the presence of TG101209-a selective JAK2 inhibitor [28]. The initial results showed that when JAK2 activity was blocked, the effects of growth inhibition and apoptosis induction of icaritin in U266 were significantly enhanced (Figure 4E, 4F). In agreement with this findings, the blocking of JAK2 by TG101209, down-regulated the expression of p-JAK2, p-STAT3, CDK2 and cyclin A, and sensitized U266 cells to the apoptosis-induction effect of icaritin (Figure 4G). These results, taken together, indicated that icaritin induced MM cells apoptosis and proliferation-inhibiting effects are mediated, in part, by JAK2/STAT3 inhibition.

### Effects of icaritin on proliferation-inhibition and apoptosis-induction in MM cells are independent of estrogen receptors blockage

To determine whether the growth-inhibiting effect of icaritin in U266 cells could be blocked by ICI 182, 780- a specific estrogen receptors antagonist, U266 cells were co-incubated with 1 μM ICI 182, 780 and various dose of icaritin (0, 2, 4, 8, 16, 32 μM) for 48 h, and then MTT assay was performed. The preliminary results showed that ICI 182, 780 interference did not abolish or influence the effect of icaritin for inhibiting the growth of U266 cells (Figure 4H). Similarly, the apoptosis-inducing effect of icaritin on U266 cells was not weakened or blocked by ICI182, 780 (Figure 4I), suggesting that the effect of apoptosis-inducing and growth-inhibition of icaritin might be independent of estrogen receptors.

### Icaritin exerts anti-myeloma activity *in vivo*

**Figure 5:**
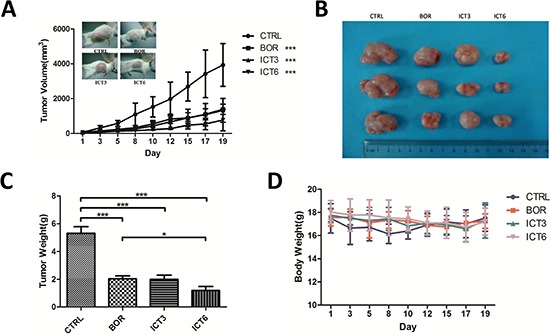
Icaritin inhibits tumor growth in xenograft mice models Six-week-old NOD/SCID mice were subcutaneously injected in the right flank area with 2 × 10^7^ cells of U266. When tumor volume reached to 50 mm^3^ after inoculation, mice (*n* = 6/group) were each given every 2–3 days intraperitionial (i.p.) injection of icaritin at 3-or 6 mg/kg in DMSO or bortezomib at 0.75 mg/kg in PBS, Control mice were given the solvent vehicle. **A.** Tumor volume was measured every other day with caliper and calculated according to the formula: *V* = 0.5 × *a* × *b*^2^. Mean ± SD, Statistical significance was determined by ANOVA, asterisks (***) represent significant (*p* < 0.001) differences relative to controls. **B and C.** Mice were sacrificed after 21 days of treatment, and the tumors were excised and weighed. The tumor weights represent the Mean ± SD. *p < 0.05; ***p < 0.001. **D.** the body weight of mice was measured on alternate days during the experiments. Results represent the Mean ± SD.

We next assessed whether icaritin could inhibit tumor growth *in vivo* with using immunoincompetent mice. U266 cells were subcutaneously inoculated into NOD/SCID mice in the right flank area. After tumors volume grew to 50 mm^3^, the mice were administered icarritin (3 mg/kg or 6 mg/kg) or bortezomib (0.75 mg/kg) every 2–3 day with intraperitoneal injection (i.p). Tumor growth and mice body weight were monitored every other day for 21 days. As show in Figure 5A, 5B and 5C, icaritin resulted in potent inhibition of tumor growth. In icaritin-treated group (6 mg/kg), the effect of icaritin on growth-inhibition was stronger than bortezomib-treated groups (Figure 5B, 5C). Moreover, body weight loss was not observed in icaritin-treated groups. At the end of experiment (the 21st day), in icaritin-treated groups, the body weight was 17.2 g ± 1.17 g, which is comparable to the control group 17.02 g ± 1.21 g (Figure 5D).

**Figure 6:**
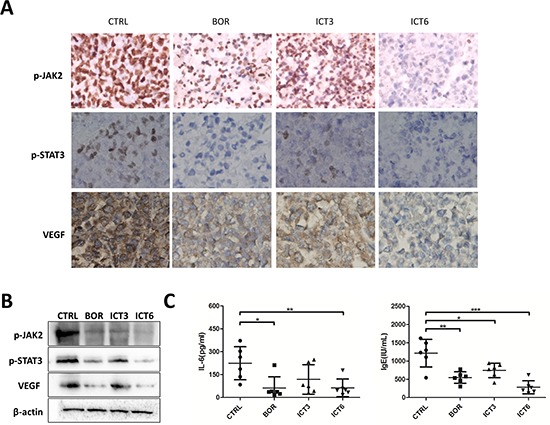
Icaritin inhibits tumor growth which corresponds to IL-6/p-JAK2/p-Stat3 reduction and decreased VEGF or IgE levels **A.** MM-bearing mice were killed on day 21, and tumors were immediately removed, fixed, embedded and sectioned at 4 μm for immunostaining of p-JAK2, p-STAT3 and VEGF, respectively. Representative photomicrograph of immunohistochemistry staining was shown (400 of magnification). **B.** Mice tumor tissue was homogenized, lysed, sheared DNA and centrifugalized, the supernatant was probed with western blot for p-JAK2, p-STAT3 and VEGF proteins. **C.** Mice serum was collected and IL-6 or IgE levels were assayed with ELISA kit.

Consistently, immunohistochemistry indicated that icaritin treatment reduced evidently the expression of p-JAK2, p-STAT3 and VEGF-angiogenesis marker compared with untreated control (Figure 6A). Corresponding to the immunohistochemistry changes, western blot analysis showed icaritin was able to down-regulate significantly the expression of p-JAK2, p-STAT3 and VEGF proteins in myeloma tissue (Figure 6B). Therefore, we may postulate that icaritin can exert anti-myeloma effects *in vivo* via suppressing “p-JAK2/p-STAT3/VEGF”-mediated signaling pathway.

More interestingly, we also observed the fact that icaritin could decrease effectively the levels of serum IL-6 and IgE in the tumor-bearing mice. As shown in Figure 6C, mouse serum IL-6 or IgE concentrations in icaritin-treated groups were significantly less with a dose-dependent manner than that of the control.

## DISCUSSION

Multiple myeloma (MM) is an incurable B-cell malignancy characterized by the accumulation of myeloma cells in bone marrow [1]. The spectrum of treatment options for multiple myeloma has dramatically changed over the past 10 years, including the application and development of new agents such as thalidomide, bortezomib, and lenalidomide. However, some multiple myeloma patients still manifest therapies-resistance and become relapsed or refractory. Recently, phytochemicals are considerably advocated as abundant sources of anti-cancer agents that are worthy of investigations. Therefore, the search for new, effective therapeutic agents, on the phytochemicals basis, for MM is urgently required.

In present study, we found icaritin, a compound purified from traditional herb medicine, has a strong anti-multiple myeloma activities towards MM cell line-U266 and primary bone marrow cells (including CD138^+^ cells) from MM patients. At concentration of 10 μM, icaritin could lead to more than 50% of growth inhibition of U266 cells. More importantly, we also found that icaritin exhibited powerful efficacies on primary bone marrow cells and CD138^+^ cells from MM patients while had not effect on proliferation of normal bone marrow cells, suggesting icaritin has low or no general cytotoxic effect on normal hematopoiesis. Consistent with this result, icaritin did not show adverse reactions such as body weight loss *in vivo* for icaritin-treated mice.

It has been established that the dysregulation of apoptosis in plasma cells is a major underlying mechanism responsible for the pathogenesis and subsequent chemo-resistance in MM [29]. Apoptosis is executed via both extrinsic and intrinsic pathways that lead to the activation of caspases [30]. Here, we showed that icaritin induced U266 cells and CD138^+^ primary MM cells apoptosis with concentration-dependent way. The regulating mechanisms for apoptosis were associated with a cleaved activation of caspase 9 and 3; up-regulated the expression of Bax, Bak proteins and down-regulated expression of Bcl-xL, which play a critical role in apoptosis by regulating the mitochondrial pathway [31]. Based on the observation, we suggested that mitochondrial-mediated caspase pathway may play a major role in icaritin-induced MM cells apoptosis.

IL-6, a multi-functional cytokine, is implicated in the development of both inflammatory diseases and tumors such as MM [32]. In MM, IL-6 is auto-secreted by myeloma cells and para-secreted mainly by bone marrow stromal cells. IL-6 binds to the sIL-6R receptor (IL-6R) on the myeloma cell surface and induces dimerization of gp130 chains, then results in activation of the associated Janus kinase (JAKs). JAKs phosphorylate gp130, leading to the recruitment and activation of the STAT3, and results in STAT3-mediated transcriptional regulation [33, 34]. In our study, we showed that icaritin significantly decreased IL-6 levels in the supernatant of cultured U266 cells, which was consistent with the changes in icaritin-treated mice serum. It has been shown that the resistance to dexamethasone in U266 cells is dependent to autocrine IL-6 [20]. More importantly, our observed that icaritin could reverse modestly the dexamethasone-resistance of U266 cells, suggesting the mechanism may be involved in the effect of icaritin for inhibiting IL-6.

Corresponding to the changes of IL-6, our data indicated that icaritin dramatically inhibited p-JAK2 and p-STAT3 expression with dose-dependent manner, and upregulated p-ERK, p-JNK levels, which may be attributed with crosstalk of different signalings, including apoptosis-related pathway under stimulated conditions. A latest study suggest that hyperactive ERK1/2 and JNK is critical to the apoptosis in anti-HLA antibody-treated MM cells [35], which is similar to our observation.

Activated STAT3 promotes tumorigenesis by blocking apoptosis and enhancing proliferation and angiogenesis, and increases cell multidrug-resistance [33, 36–39]. In our study, we found that icaritin evidently inhibited IL-6/STAT3 activities, associated with upstream p-JAK2 inhibition. To further demonstrate the significance of JAK2 and STAT3, we utilized JAK2 inhibitor -TG101209 and STAT3 siRNA to block the activity of JAK2 and the expression of STAT3 respectively. As shown above, the inhibition of JAK2 or signal blocking of STAT3 did not abolish completely the effect of icaritin on growth-inhibiting and apoptosis-inducement of U266 cells. Several key signaling factors, which are responsible to growth/apoptosis regulation, including cyclin A or B, CDK2, cyclin E and caspase 3, were also reduced or activated by icaritin treatment, suggesting although icaritin may potently inhibits the JAK2/STAT3 signaling axis in U266 cells, the crosstalk or inhibition of other signal pathways, such as cell cycle-regulated or apoptosis signalings, obviously was involved in the mechanisms of icaritin for anti-myeloma activity. Therefore, the interruption of JAK2/STAT3 signaling is the main molecular event for the effect of icaritin against MM, not only molecular target.

It has been reported that constitutive activity of STAT3 up-regulated VEGF expression and tumor angiogenesis [40]. MM cells are capable of secreting VEGF in response to IL-6 stimulation, and contribute to the growth and survival of malignant plasma cells [41]. Consistently, we confirmed anti-myeloma effect of icaritin in human primary MM xenograft mouse model by down-regulating the levels of p-JAK2, p-STAT3 and VEGF. As shown above, icaritin suppressed the expression of p-JAK2, p-STAT3 as well as VEGF in myeloma tissue evaluated by immunohistochemistry and western blot, which were consistent with the results of *in vitro* experiments. Furthermore, we also found that icaritin was able to significantly decrease the levels of serum IL-6 and IgE in myeloma-bearing mouse, supporting that icaritin plays a critical role in anti-myeloma effect by inhibiting the activation of p-JAK2, p-STAT3, down-regulating the expression of VEGF and reducing the secretion of IL-6.

Although icaritin possesses estrogen-like activity and functions as an estrogen receptor modulator for regulating cell growth, in the current study, we did not confirm that the anti-proliferation activity of icaritin on U266 cells was dependent on the activation or blocking of estrogen receptor. Actually, ICI 182, 780 blocking test had revealed that even if estrogen receptor on U266 cells was blocked by ICI 182, 780, it did not lead to the growth arrest or weaken the effects of icaritin for proliferation-inhibition of U266 cells.

In conclusion, we have documented for the first time the anti-MM effects of icaritin *in vitro* and *in vivo*. Our findings have highlighted the fact that icaritin is able to inhibit MM cells growth, induce apoptosis and no general cytotoxic effect. The underlying mechanisms of icaritin anti-MM activity are mainly involved in the inhibition of IL-6 driven-JAK2/STAT3 signaling pathway, and in part associated with the crosstalk and inhibition of other growth-related signals. Our study indicates icaritin as a natural product in treating refractory MM, provides a new approach and alternative choice.

## MATERIALS AND METHODS

### Cell line and reagents

Human multiple myeloma cell line U266 (ATCC TIB-196) was maintained in RPMI-1640 medium containing 10% heat-inactivated fetal calf serum, 2 mM L-glutamine, penicillin-streptomycin (100 U/mL and 100 U/mL, respectively). Icaritin with a purity of up to 99.5% was provided by Dr. Kun Meng (Shenogen Phama Group, Beijing, China). A stock solution (32 mM) was prepared by dissolving icaritin in DMSO (Sigma, St.Louis, MO, USA) and storied at −20°C. Human CD138 MicroBead was purchased from Miltenyi (Miltenyi Biotec GmbH, Germany). Antibodies for Bax, Bak, Bcl-xL, caspase 9, cyclin A and β-actin were purchased from Santa Cruz Biotechnology (Santa Cruz, CA); Antibodies for caspase 3, JNK, ERK1/2, STAT3, phospho-JNK, phospho-ERK, phospho-STAT3, CDK2, cyclin B were from Cell Signaling Technology; PE-conjugated anti-CD138 antibody were from BD; anti-VEGF and anti-phospho-JAK2 antibodies, Fulvestrant (ICI 182, 780) and Human IgE ELISA assay kit were purchased from Abcam (Hong Kong) Ltd. Human IL-6 ELISA assay kit was from R&D Systems China CO., Ltd. MTT (3-(4, 5-dimethylthiazol-2-yl)-2, 5-diphenyl-tetrazolium bromide) was dissolved in PBS and stored at −20°C.

### Isolation of primary myeloma cells and purification of CD138^+^ MM cells

Primary MM cells were collected from bone marrow samples of patients with MM and bone marrow mononuclear cells (BMMCs) were isolated by Ficoll-Paque isolation solution. The CD138^+^ cells of bone marrow were isolated and purified by CD138 selection kit. MM was diagnosed according to WHO criteria [42]. Total of 35 MM patients were enrolled into the study. 11 of normal bone marrow samples were used as controls. Among the patients, BMMCs were isolated from 28 MM patients; CD138^+^ cells were purified as described in ref [43] in 14 MM patients. All patients and normal controls provided written informed consent for the collection of samples and subsequent analysis. This study was conducted according to the Declaration of the Helsinki, and approved by the Institutional Review Board of the Second Xiang-Ya hospital, Central South University.

### Cell culture and cytotoxicity assay

U266 cells, BMMCs and CD138^+^ cells derived from MM patients were maintained in RPMI-1640 medium (Gibco) supplemented with 10% FBS and antibiotics. The cells were treated with various concentrations of icaritin (0 μM; 2 μM; 4 μM; 8 μM; 16 μM, 32 μM) for 48 hours. The cytotoxic effect of icaritin was evaluated by MTT method [10]. For time courses analysis, U266 cells were treated with indicated icaritin concentrations for 24 h, 48 h, and 72 h, respectively. Cell viability was calculated as a percentage of viable cells in icaritin-treated group versus untreated control.

### Apoptosis assay

U266 Cells or CD138^+^ cells from MM patients (3 × 10^5^/ml) were seeded in 6-well plate and incubated with different concentration of icaritin as indicated-above for 48 hours. The morphologic change of apoptosis for MM cells was evaluated by Wright-Giemsa staining under light microscope. Early apoptosis were assessed with Annexin V-FITC/propidium iodide(PI) apoptosis detection Kit (Becton Dickinson, BD, USA) combined Flow cytometry (FACS-Caliber, BD, USA).

### Cell cycle analysis

U266 Cells were treated for 48 h with various concentrations of icaritin. The cells were harvested, washed with ice-cold PBS, fixed with 70% cold ethanol for overnight and pretreated with 10 ug/mL of RNAse for 30 minutes. Cells were stained with propidium iodide (Sigma Chemical). The cell-cycle profiles were determined by using ModFit LT 3.0 software packages on FACS-Calibe flow cytometry.

### Western blotting

U266 cells were treated with various dose of icaritin for 48 hours, harvested, washed, and lysed using lysis buffer (Pierce® IP lysis buffer, Thermo, USA) containing 2 mM Na_3_VO_4_, 1 mM NaF and 1 mM phenylmethylsulfonyl fluoride. After determining protein concentration, equal amount of protein (~50 μg/well) was separated on 8–15% SDS-PAGE and transferred onto nitrocellulose membranes. The membranes were then probed with various primary antibodies, HRP-conjugated secondary antibodies, and visualized with enhanced chemiluminescence (ECL) detection kit (Amersham Pharmacia Biotech).

### ICI 182, 780 blocking experiment for estrogen receptor

The effects of phytoestrogens are mediated through two well-characterized intracellular receptors-estrogen receptor (ER) α and β. ERs are members of the nuclear receptor superfamily and act as a ligand-activated transcription factors to regulate the expression of target genes [44, 45]. To determine if the effect of icaritin against MM activities is dependent on estrogen receptor α or β, we carried out a blocking experiment using ICI 182, 780, a specific estrogen receptor antagonist. After treating U266 cells for 4 hours with ICI 182, 780 (1 μM) [12], U266 cells were exposed to various dose of icaritin (0, 2, 4, 8, 16, 32 μM) for 48 h, the cytotoxic assay (MTT) and apoptosis identification (Annexin V assay) were performed as above-mentioned methods.

### Enzyme-linked immunosorbent assay (ELISA) for IL-6 and IgE levels

IL-6 levels were measured in supernatant from cultured U266 cells and in serum from MM xenograft mouse with a commercially available human IL-6 ELISA kit according to the manufacture's protocols. The range of detection was 3.12 pg/ml to 300 pg/ml. Because U266 cells were characterized by secreting monoclonal IgE. Mouse serum IgE levels from the MM xenograft mice were detected using human IgE ELISA kit following manufacture's instruction.

### SiRNA interference for STAT3

2 × 10^5^ U266 cells were plated onto a 6-well culture plate in 2 ml complete medium, siRNA oligonucleotides targeting STAT3 gene (CCGTGGAACCATACACAAA) and negative control siRNA (Guangzhou Ribobio CO., LTD, China) were transfected at a final concentration of 50 nM by using ribo FECT^TM^ CP transfection reagent (Guangzhou Ribobio CO., LTD, China). After 24 hours, the cells were exposed to DMSO or icaritin (16 μM) for 48 hours, cells were collected and cellular proteins were extracted for western blotting assay.

### MM xenograft mouse models

Female NOD/SCID mice (6-week old) were purchased from Vital River Laboratories (Beijing, China) and maintained under conventional conditions. All animal work was approved by the Institutional Review Board of the second Xiangya hospital, Central south University. U266 cells (2 × 10^7^ cells) were injected into each mouse by subcutaneously inoculation in the right flank area. After tumors volume grew to 50 mm^3^, the mice were administered icaritin (3 mg/kg or 6 mg/kg) or bortezomib (as positive control; 0.75 mg/Kg) every 2–3 day with intraperitoneal injection (i.p). Tumor growth and mice body weight were monitored every other day for 21days. Tumor volume was calculated using the formula: *V* = 0.5 × *a* × *b*^2^, where a and b represented the long and short diameter of the tumor, respectively. At the twenty-first day, all mice were sacrificed individually by cervical dislocation and blood was collected for ELISA. Tumor xenografts were removed, weighed, incised and pathological sections were prepared and stained with immunohistochemistry.

### Immunohistochemistry

Paraffin-embedded tissues were sectioned 4 μm thick slices, DAB detection kit (Fouzhou maxim Biotechnology Company, China) was used for immunohistochemical staining, according to the manufacturer's instruction. Briefly, p-JAK2 primary antibody (1:100), p-STAT3 primary antibody (1:100), VEGF primary antibody (1:200) and biotin-labeled secondary antibody 100 μl was added. The slices were visualized for 3 minutes following staining, then re-stained with hematoxylin and sealed. Positive cells were stained into brown. The staining intensity and positive ratio of the mentioned proteins were observed under microscope by two independent scorers blinded to the clinical parameters.

### Statistical analysis

All values were expressed as means ± standard deviation (SD) for the indicated number of separate experiments. Statistical analysis of multiple-group comparisions was performed by one-way analysis of variance (ANOVA). Comparisions between two groups were analyzed using 2-tailed Student *t*-test. All statistical analyses were performed by using spss 17.0 for window (Chicago, IL, USA). Statistical significance was defined as a *P* value < 0.05.
